# Ultrasonication-Induced Preparation of High-Mechanical-Strength Microneedles Using Stable Silk Fibroin

**DOI:** 10.3390/polym16223183

**Published:** 2024-11-16

**Authors:** Huihui Liang, Jiaxin Chen, Guirong Qiu, Bohong Guo, Yuqin Qiu

**Affiliations:** 1 Department of Pharmaceutics, School of Pharmacy, Guangdong Pharmaceutical University, Guangzhou 510006, China; 17703019860@163.com (H.L.); m13924254823@163.com (J.C.); qiugr0602@163.com (G.Q.); 2 Guangdong Provincial Key Laboratory for Research and Evaluation of Pharmaceutical Preparations, Guangdong Provincial Engineering Center of Topical Precise Drug Delivery System, Guangdong Pharmaceutical University, Guangzhou 510006, China

**Keywords:** silk fibroin, microneedles, ultrasonication, stability, mechanical strength

## Abstract

Silk fibroin (SF) is an ideal material for microneedle (MN) preparation. However, long extraction and short storage durations limit its application. Furthermore, MNs prepared from SF alone are easy to break during skin insertion. In this study, a regenerated SF solution was autoclaved and freeze-dried to produce a stable and water-soluble SF sponge. The freeze-dried SF (FD-SF) solution was ultrasonically treated before being used in the fabrication of MNs. The ultrasonically modified SFMNs (US-SFMNs) were evaluated in comparison to FD-SFMNs made from FD-SF and conventional SFMNs made from regenerated SF. The results indicated that the FD-SF could be completely dissolved in water and remained stable even after 8 months of storage. FTIR and XRD analyses showed that SF in US-SFMNs had increased β-sheet content and crystallization compared to FD-SFMNs, by 7.3% and 8.1%, respectively. The US-SFMNs had higher mechanical strength than conventional SFMNs and FD-SFMNs, with a fracture force of 1.55 N per needle and a rat skin insertion depth of 370 μm. The US-SFMNs also demonstrated enhanced transdermal drug delivery and enzymatic degradation in vitro. In conclusion, the autoclaving and freeze drying of SF, as well as ultrasonication-induced MN preparation, provide promising SF-based microneedles for transdermal drug delivery.

## 1. Introduction

Silk fibroin (SF), a protein extracted from silkworm cocoons, has been widely employed in various biomedical fields, such as wound dressing, tissue engineering, and drug delivery [1]. Microneedles (MNs) are a new transdermal technology that have grown in popularity in recent years due to their ability to provide the benefits of both a transdermal patch and a hypodermic injection. SF’s unique mechanical properties, biodegradability, and biocompatibility made it an idea option for MN fabrication [2,3,4,5]. Silk-fibroin-based microneedles (SFMNs) have been used in a variety of applications, including wound healing [6,7], scar repair [8,9], and diabetic management [10,11,12,13]. Many studies have demonstrated that SFMNs can achieve results comparable to or greater than those of needle injection. Even with these promising findings, more research is required to hasten the introduction of SFMNs into the medical market.

The first concern is the shortage of stable SF material for the formulation development and mass production of SFMNs. As an excellent biomaterial, SF can be used to make MNs under mild processing conditions. Most studies began with the extraction of SF from silkworm cocoons [14,15,16]. The typical procedure consists of degumming (sericin removal), washing, drying, dissolving fibroin fibers, and dialysis of the dissolved fibroin solution. The entire procedure takes 4 days or longer. Moreover, the resulting SF solution can be refrigerated for no more than 7 days. Previous research has shown that not only does it take a long time to extract SF from silkworm cocoons, but extraction from transgenic spiders also requires procedures such as SF purification and concentration, resulting in a long time cycle and stability issues [17]. The long extraction and short storage durations for the regenerated SF solution greatly impair the preparation efficiency of SFMNs. Freeze drying is a common approach for increasing the stability of biomacromolecules. However, directly freeze drying an SF solution produces solid SF that is insoluble in water and cannot be reconstituted into an SF solution for use in preparing MNs. As a result, the regenerated SF solution requires a particular treatment to ensure long-term stability for further SFMN preparation.

On the other hand, despite the fact that SF has superior mechanical properties to many other natural polymers such as collagen and polysaccharides [18,19], conventional SFMNs prepared from SF alone are easy to break [20]. SFMNs must be modified to improve their mechanical strength, which is related to the conformation of SF. In its solid state, SF exists in three main conformations: random coil, Silk I, and Silk II [21,22]. The crystalline form of SF is represented by Silk I and Silk II. Silk II’s antiparallel β-sheet layered structure makes it stable and insoluble in water. The random coil conformation is unstable and can convert into β-sheet when exposed to specific environmental stimuli, including changes in pH, humidity, and temperature. The β-sheet enhances the mechanical strength of SF materials by offering physical cross-linking points through intermolecular force and hydrogen bonding [2]. Certain research has suggested that increasing the β-sheet content in SF by methanol, ethanol, or water vapor annealing can enhance its mechanical strength [22,23,24]. However, annealing SFMNs following MN preparation may result in a poor shape, and the presence of methanol and ethanol residues make SFMNs less biocompatible. Therefore, alternative methods are required to increase the mechanical strength of SFMNs.

Furthermore, the current regulatory approval procedure for microneedle patches is not ideal because of the advanced technology. According to FDA guidance for microneedle products (Regulatory Considerations for Microneedling Products, Document issued on 10 November 2020), safety data should be obtained to support the safe usage of MNs. Safety is the primary obstacle to the commercialization of SFMNs, according to a review by Qi et al. [5]. Despite the widespread recognition of SF’s biosafety, fundamental research, such as biodegradation testing, is still required. Standardized regulation of sterilizing techniques is also necessary for applications in the future market.

In this study, to prepare stable and water-soluble SF material, an SF solution extracted from silkworms was autoclaved before freeze drying. The freeze-dried SF (FD-SF) sponge was then characterized using redissolution experiments and gel permeation chromatography (GPC), scanning electron microscopy (SEM), Fourier transform infrared spectroscopy (FTIR), and X-ray diffraction (XRD) analyses. To improve the mechanical strength of the SFMNs, the FD-SF solution was ultrasonicated before MN preparation, drawing inspiration from research using ultrasonication to induce the sol–gel transition of SF [25,26,27]. The ultrasonically modified SFMNs (US-SFMNs) were evaluated for SF structure, mechanical strength, insertion ability, and swelling and dissolving properties. The in vitro enzymatic degradation of US-SFMNs and transdermal delivery of a model drug, colchicine, were also investigated.

## 2. Materials and Methods

### 2.1. Materials

*Bombyx mori* silkworm cocoons were provided by Northwest Silkworm Company (Shanxi, China). Sodium carbonate (Na_2_CO_3_) and sodium bicarbonate (NaHCO_3_) were obtained from Tianjin Damao Chemical Reagent Factory (Tianjin, China). Lithium bromide (LiBr) was obtained from Shanghai Aladdin Bio-Chem Technology Co., Ltd. (Shanghai, China). PEG-20000 was obtained from Tianjin Damao Chemical Reagent Factory (Tianjin, China). Protease XIV was purchased from Shanghai Yuanye Biotechnology Co., Ltd. (Shanghai, China). Colchicine was purchased from Meryer Biochemical Technology Co., Ltd. (Shanghai, China).

Chinese male SD rats (180–200 g) were obtained from the Laboratory Animal Management Center of Southern Medical University (Guangdong, China). The rats were housed under special pathogen-free conditions. All animal experiments followed the National Institutes of Health Guide for the Care and Use of Laboratory Animals (8th edition). The animals involved in this study were approved by the Experimental Animal Center of Guangdong Pharmaceutical University and followed the National Institute of Health and Nutrition Guidelines for the ethical use of animals.

### 2.2. Extraction of Silk Fibroin

The SF was extracted from natural silkworm cocoons as reported previously [28]. Briefly, the cocoons were boiled in a Na_2_CO_3_/NaHCO_3_ solution (0.01 M, pH = 9) for 30 min before being thoroughly washed three times with deionized water (DI water). The silk fibers were then dried at 60 °C, and after that, they were dissolved in 100 mL of LiBr solution (9.3 M) at 60 °C for 2 h. After cooling and dialyzing the solution for 24 h against DI water, it was concentrated using reverse dialysis against a 20% PEG-20000 solution. Before being used, the resulting SF solution was kept in a refrigerator at 4 °C. Weighing a particular volume of the solution after drying allowed us to determine the final concentration of the SF solution.

### 2.3. Autoclaving and Freeze Drying of Silk Fibroin

#### 2.3.1. Preparation of Freeze-Dried Silk Fibroin (FD-SF) Sponges

Sterilization is a fundamental requirement for parenteral preparations [29]. In this study, the SF solution was sterilized using autoclaving before being freeze dried. After diluting the SF solution to 3 wt% in DI water, it was autoclaved for 20 min at 121 °C using a high-pressure saturated steam cycle (3 mL/test in 8 mL glass vials protected with aluminum foil). Following autoclaving, the SF solution was pre-frozen at −20 °C for 8 h then lyophilized for 48 h to remove the water and form FD-SF sponges. The sponges were sealed and protected from light in a refrigerator.

#### 2.3.2. Redissolution of the FD-SF Sponges

The FD-SF sponges with weights of M_1_ were submerged in 5 mL of DI water, the redissolution process was observed, and they were photographed at 0.5, 1, and 4 h. The solutions were centrifuged at 10,000 rpm for 20 min. After being carefully pipetted out, the supernatants were dried at 60 °C until they reached a constant weight. Following drying, the weight was noted as M_2_. Eventually, the redissolution ratio was calculated according to the following equation:

(1) $$Redissolution\;Ratio\left(\%\right)=\frac{M_{2}}{M_{1}}\times 100\%$$

#### 2.3.3. Scanning Electron Microscopy (SEM)

The FD-SF sponges were cut into thin slices with Gillette blades, and the cross-section surface of the FD-SF sponges was examined by SEM (SU8010, Hitachi, Tokyo, Japan). The samples were examined by SEM under an accelerating voltage of 3.0 kV after being coated with gold using a sputter coater.

### 2.4. Molecular Weight (MW) Distribution of SF

The MW distributions of SF in the regenerated SF solutions and redissolved FD-SF solutions were measured by gel permeation chromatography (GPC; 1525, Waters, MA, USA), and a TSKGgel G3000SWXL column (Tosoh, Tokyo, Japan) was used as the solid phase. A 20 μL solution was injected for each sample, with a 0.1 M sodium sulfate, 0.1 M sodium phosphate buffer solution (pH 6.7) flowing at 0.5 mL/min at 30 °C as the mobile phase. The protein content was determined by measuring UV absorbance at 280 nm. The MWs were measured after obtaining a calibration curve using cow thyroglobulin, bovine hemoglobulin, and other reference standards.

### 2.5. Ultrasonication of FD-SF Solution

An amount of 5 mL of the FD-SF solutions (8 wt%) was sonicated with the power of 100 W for 10, 20, and 30 min using a cell ultrasonic pulverizer (Scientz-IID, Xinzhi, Ningbo, China) equipped with a 6 mm diameter microtip. The solutions were kept at 4 °C during sonication. The particle size distribution of the SF, FD-SF, and ultrasonicated silk fibroin (US-SF) solutions was measured using a dynamic light scattering spectrophotometer (Micro-Nano Winner 802, Winner, Jinan, China).

### 2.6. Preparation of MNs

The regenerated SF, FD-SF, and US-SF solutions with an SF concentration of 8 wt% were prepared. An appropriate amount of colchicine was dissolved in the solutions, if added. After casting 200 μL of each solution into the PDMS mold and vacuuming it for 30 min, the mold was kept left overnight at room temperature in a dehumidifier. Finally, conventional SFMNs, FD-SFMNs, and US-SFMNs were demolded and stored at room temperature until use. MNs prepared from the US-SF solution, which was ultrasonicated for 10 min, 20 min or 30 min, were recorded as US-SFMNs-10 min, US-SFMNs-20 min, and US-SFMNs-30 min, respectively.

### 2.7. FTIR and XRD Analyses

Each natural protein has a unique 3D structure, known as its conformation, which determines its capacity to fulfill a certain function. In practical research, the structure of SF is typically classified into Silk I and Silk II crystalline forms using XRD. And FTIR is commonly used to analyze the secondary structure of SF, using random coils, α-helices, β-sheets, β-turns, and side chains. In the process of silk spitting by a silkworm, with the loss of water and concentration of the SF solution, the SF is transformed from a random coil structure to a Silk I structure, which is then transformed into a Silk II structure. The structure of Silk II’s crystalline form has received considerable attention because of its early discovery. It is well known that Silk II exists as an antiparallel β-folded structure [30], while the structure of Silk I is somewhat controversial. Previous researchers concluded that Silk I’s structure contained mainly α-helices and random coils, but recently, researchers showed that Silk I was a type-II β-turn structure using the 2-D NMR technique [31].

An FTIR analysis of the FD-SF sponges before and after storage, conventional SFMNs, FD-SFMNs, and US-SFMNs was performed using a Thermo Nicolet iS5 spectrometer with an attenuated total reflectance (ATR) attachment (Nicolet iS5, Thermo, Waltham, MA, USA) for multiple reflection. The wavenumber was in the range of 500−4000 cm^−1^. To identify secondary structures in protein samples based on absorption spectra, the peak positions of the amide I region (1605–1703 cm^−1^) absorption were obtained from Fourier self-deconvolution (FSD) using OMNIC software 9.2.86 (MicroCal, Northampton, MA, USA), as previously published [32]. The peak absorption bands were assigned as shown in the Supplementary Materials (Table S1) [33].

An XRD analysis was carried out to understand the aggregation structure of the SF samples using an X-ray diffractometer (XRD Ultima IV, Rigaku, Tokyo, Japan). The XRD patterns were recorded in the range of 2*θ* from 5 to 45° at a 10° min^−1^ speed, 40 kV, and 35 mA. The crystallinity of the SF samples was calculated by a similar FSD process using MDI Jade software 6.5.26 (Materials Data, Livermore, CA, USA).

### 2.8. Swelling and Dissolving Capacities of Microneedles

The MNs were weighed (4 parallel samples in each group) and denoted as “M”. After that, the MNs were submerged in PBS (pH = 7.4) at a bath ratio of 1:100 (*w*/*v*) for 24 h at 37 °C. After soaking, the surface moisture of the MNs was removed, and the swollen MNs were weighed and marked as “M_1_”. The MNs were then dried in an oven to a consistent weight, and the final weight was noted as “M_2_”. The morphological dimensions of MNs prior to and following swelling were monitored by a digital camera. The swelling and dissolving ratio were calculated according to the following equations:

(2) $$Swelling\;ratio\left(\%\right)=\frac{M_{1}}{M_{2}}\times 100\%$$

(3) $$Dissolving\;ratio\left(\%\right)=\frac{M-M_{2}}{M}\times 100\%$$

### 2.9. Mechanical Strength and Insertion Ability of Microneedles

A texture analyzer (TA-XT plus, PerkinElmer, Waltham, MA, USA) was used to assess the mechanical strength of the SFMNs using a compression test to evaluate their use. The parameters that were used in the testing for each type of MN are listed in the Supplementary Materials (Table S2). After positioning the MNs with their tips facing up on the operating platform, they were compressed at a continuous rate of 6 mm/min until the needles buckled or broke. Measurements were taken of the compression force (N) versus displacement (mm).

The ex vivo insertion capacity of the SFMNs was studied using rat skin. The tests were conducted using the abdominal skin of male Sprague-Dawley rats weighing 180–220 g. Excessive ethylether anesthesia was used to sacrifice rats. A pair of scissors was used to delicately trim the rat’s hair short, and the skin around its abdomen was cut off. Next, a scalpel and isopropyl alcohol were used to remove the fat that had adhered to the dermis side. The resulting skin was cleaned with a 0.9% sodium chloride solution, sectioned into appropriate-sized pieces, and stored at −80 °C (no longer than 2 months) until use. A piece of an MN patch was placed against the rat skin using 40 N of force for 30 s. After the MNs were removed from the skin, a cotton swab was used to clean the treated area. The skin treated with MNs was then fixed in 4% paraformaldehyde for more than 24 h. After that, the skin was fixed in paraffin, sliced longitudinally, and stained with hematoxylin–eosin (H&E). Micropores in skin slices were observed using a microscope (EVO MA 10, Carl Zeiss, Jena, Germany).

### 2.10. Enzymatic Degradation of SFMNs

The conventional SFMNs, FD-SFMNs, and US-SFMNs were incubated at 37 °C in PBS (pH = 7.4) containing 2 U/mL protease XIV with a bath ratio of 1:100 (*w*:*v*). The solution for degradation was renewed daily. At predefined intervals, samples were washed with DI water and dried to constant weight after collection through centrifugation, with 4 parallel samples each group.

### 2.11. In Vitro Transdermal Delivery of Colchicine from SFMNs

To evaluate the transdermal delivery characteristics of the conventional SFMNs, FD-SFMNs, and US-SFMNs, a first-class antigout drug, colchicine, was used as the model drug, as it was investigated in our earlier study [28]. The permeation of colchicine from the conventional SFMNs, FD-SFMNs, and US-SFMNs over rat skin was assessed using Franz diffusion cells. The rat skin was cut into pieces that were somewhat bigger than the donor. The MNs were then punctured into the skin for 30 s using a force of 40 N while being secured with a surgical patch. After that, the skin was fixed between the diffusion cells’ donor and receptor compartment, with MNs attached to it. PBS (pH = 7.4) was added to the receptor compartments, and they were agitated at 300 rpm and 37 °C. Samples were taken out of the receptor compartment and replaced with an equal volume of fresh PBS at prearranged intervals. The colchicine concentration in the samples was measured using HPLC. At 1, 2, 4, 6, 8, 12, and 24 h, the samples were taken out. Following that, 0.22 μm microfiltration membranes were used to filter the samples. Twenty microliters of filtrate were injected into a high-performance liquid chromatography (HPLC) system. The mobile phase used for the analysis was acetonitrile–water (35:65, *v*/*v*), and the flow rate was set at 1.0 mL/min. At room temperature, a Diamonsil C18 chromatographic column (4.6 mm × 250 mm, 5 μm) was used, with a 349 nm detection wavelength. The regression equation y = 65.878x + 11.206 (r = 0.9998) was used to calculate the concentrations of colchicine. In this equation, “y” stands for the sample’s peak area as determined by HPLC and “x” for the colchicine concentration in the solutions.

### 2.12. Statistical Analysis

A statistical analysis was carried out with SPSS 19.0 software. Data are reported as mean ± standard deviation (SD). To compare the experimental groups in every scenario, *t* tests were employed, with *p* < 0.05 indicating statistical significance.

## 3. Results and Discussion

### 3.1. Autoclaving and Freeze Drying of SF Solutions

The topic of whether MNs need to be sterile or not is debatable [34]. According to certain research, MNs have less microbial penetration than hypodermic needles, implying a lower infection risk. There is also evidence that infection remains a potential concern, with erythema, pain, oedema, and skin irritation as side effects [35]. Owing to the parenteral administration of MNs, it is still possible that regulatory authorities will need guarantees of absolute sterility for MN products [36]. The FDA recommends that sterilization be considered as early as possible in the development process. Commonly used moist and dry heat sterilization have shown to be harmful to MN devices [36]. Gamma irradiation can also reduce SF content and possibly cause SF damage [37]. For some specialized MNs like SF-based MNs, aseptic production may be the only viable option. As a result, sterile MN material will be required. In this study, the SF solution was autoclaved before freeze drying rather than after freeze drying, because previous research has demonstrated that autoclaving of lyophilized SF sponges decreased the scaffold degradation rate in vitro [37]. The results showed that the SF solution was slightly yellowish after autoclaving. The autoclaved solution was then lyophilized, as was the non-autoclaved solution, which served as the control. Except for a minor yellowing after sterilization, there was no significant difference between the freeze-dried SF sponges (FD-SF) with and without sterilization.

### 3.2. Redissolution and Molecular Weight Determination of Freeze-Dried SF Sponges

**Figure 1:**
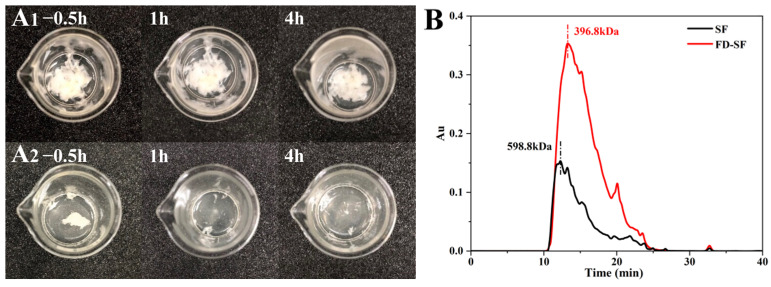
(**A**) Redissolution of non-autoclaved (**A1**) and autoclaved (**A2**) SF lyophilized sponge; (**B**) molecular weight of freeze-dried SF (FD-SF) and regenerated SF (SF) determined by GPC.

The FD-SF needed to be reconstituted into FD-SF solutions for MN preparation. Therefore, the ideal FD-SF sponge should be capable of complete and rapid resolubilization. Two samples of 0.4 g of FD-SF, with and without autoclaving, were dissolved in 5 mL of deionized water each. The redissolution ratio was then calculated by determining the SF amount in the solution. Figure 1A shows FD-SF dissolved for 0.5, 1 and 4 h. After 0.5 h of immersion in DI water, more than half of the autoclaved FD-SF had dissolved, with the majority dissolving by 1h, and there was no insoluble material after 4 h. In contrast, the non-autoclaved FD-SF was rarely dissolved even after 4 h of immersion in DI water. The final redissolution ratio of the autoclaved FD-SF was 99.6%, while the non-autoclaved FD-SF was only 60%. The results demonstrated that FD-SF with sterilization was more soluble in water than FD-SF without sterilization. Autoclave sterilization before freeze drying can not only result in sterilized SF but also achieve rapid and complete redissolution of the FD-SF for MN preparation.

Gel permeation chromatography (GPC) was utilized to determine the molecular weight of SF in the regenerated SF solution and FD-SF solution. As shown in Figure 1B, the molecular weight of SF in the regenerated solution was 358.7 kDa. Variations in degumming periods and procedures can result in different molecular weights of SF [38,39]. Our findings are comparable to Cho et al.’s study [40]. In contrast, FD-SF has a molecular weight of 266.8 kDa, indicating that the molecular weight of SF decreased after autoclave sterilization and freeze drying. According to Kovacina et al.’s research, autoclaving the SF solution lowered its average molecular weight, as determined by SDS-PAGE and Coomassie staining, since autoclaving can cause considerable fragmentation of fibroin chains, resulting in a reduction in molecular weight [37]. One of the reasons why the autoclaved FD-SF redissolved completely and quickly could be its lowered molecular weight.

### 3.3. SEM Characterization

**Figure 2:**
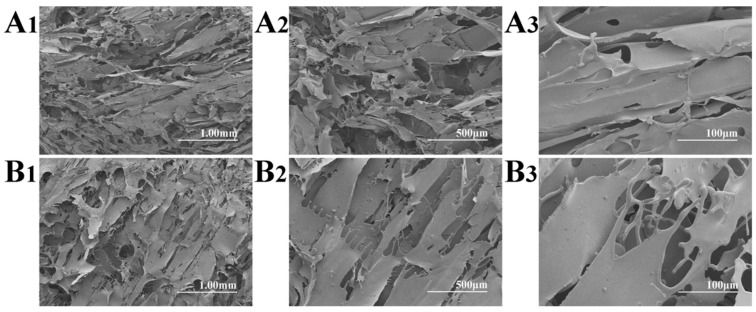
SEM micrographs of non-autoclaved (**A1**–**A3**) and autoclaved (**B1**–**B3**) FD-SF.

Figure 2 shows the SEM micrographs of the non-autoclaved and autoclaved FD-SF. For the FD-SF that was prepared without autoclaving, an interconnected lamellar structure was observed (Figure 2A1–A3), which was consistent with the previous research findings [31]. As shown in Figure 2B1–B3, when the FD-SF was autoclaved before freeze drying, a lamellar morphology could still be seen, with a loose structure and larger pores, which may have contributed to the FD-SF’s redissolution capacity.

### 3.4. Stability of Freeze-Dried SF Sponges

One of the most important problems regarding SFMN preparation is the instability of the regenerated SF solution, which is prone to gelling, as a gelatinized SF solution is unsuitable for preparing MNs due to its low fluidity. The rate of gelation accelerates with increasing temperature and SF content [41]. In this study, the stability of the SF solution with 8% concentration, which was suitable for MN preparation in our earlier study, was investigated under refrigerated storage (4–8 °C). It was found that the solution became viscous and had difficulty flowing after only one day of storage. And on the fifth day, the solution had totally transitioned into gel. Therefore, freeze drying SF solutions is important since the SF extraction is time consuming and the solution has a short preservation duration [42]. More crucially, by autoclaving SF before freeze drying, we were able to resolve the problem of the freeze-dried SF being insoluble in water. The autoclaved FD-SF was sealed and refrigerated, taken out after 8 months and found to be unaffected in color and shape. To prepare MNs, the solid SF material must be redissolved in water. The redissolution ratio of the FD-SF after storge was demonstrated to be 97.5 ± 0.87%, comparable to the freshly prepared FD-SF.

**Figure 3:**
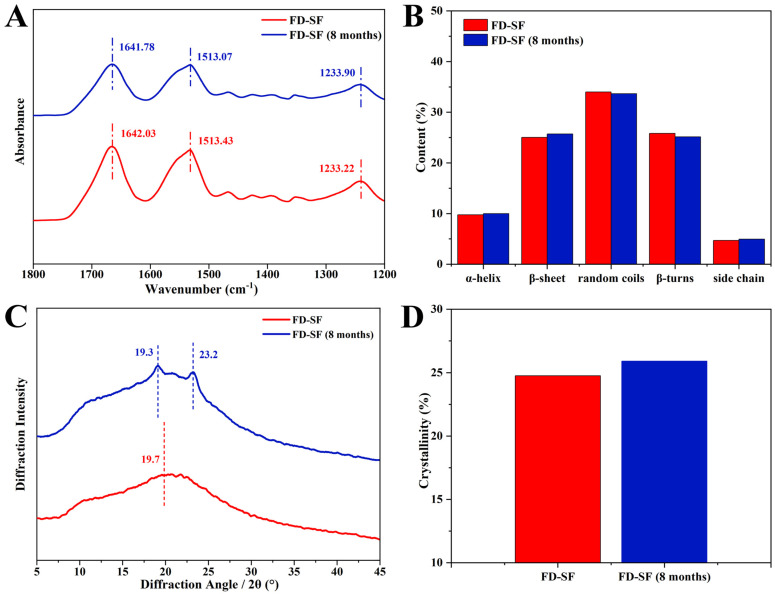
Structure analysis of FD-SF before and after 8 months of storage: (**A**) FTIR spectrum; (**B**) secondary structure content of FD-SF; (**C**) X-ray diffraction curve; (**D**) crystallinity of FD-SF.

The stability of the FD-SF was further investigated using FTIR and XRD analyses. FTIR spectroscopy is a powerful method for analyzing protein secondary structure. In FTIR, the sections that characterize SF are typically amide I, amide II, and amide III. The amide I region is largely caused by the overlap of stretching vibrations of the C=O group in the protein backbone [33]. The amide II region band is slightly weaker than the I region and represents the stretching of the N-H bond, whilst the amide III region band is often the least noticeable and represents a combination of vibrations such as alkyl and tyrosine [43]. Among these, the amide I region at 1605–1703 cm^−1^ can be used to attribute the secondary structure of proteins and investigate their internal hydrogen bonding, and hence are the absorption bands of focus. The distribution of vibrational bands in the amide I region of the secondary structure of SF is shown in the Supplementary Materials (Table S1) [33]. As shown in Figure 3A, when the FD-SF had been stored for 8 months, the characteristic FTIR peaks varied very little, and the peak areas remained nearly unchanged. Figure 3B shows the fraction of each secondary structure determined by Fourier self-deconvolution (FSD). After 8 months of storage, the variation between each secondary structure was less than 1%. Details of the FSD process are shown in the Supplementary Materials (Tables S3 and S4). Since the secondary structure of proteins is a key factor in determining their properties, the FTIR data demonstrated that the FD-SF could be stable in the refrigerator for 8 months.

Further characterization using XRD investigated the change in crystallization in the FD-SF following storage. The main diffraction peaks at 12.2°, 19.7°, 24.7°, 28.2°, 32.3°, and 36.8° represent Silk I, while those at 9.1° and 20.7° represent the Silk Ⅱ structure [14,44]. As shown in Figure 3C, the freshly prepared FD-SF had an amorphous structure with a widespread peak at 19.7° in the XRD curves. Crystal peaks at 19.3° and 23.2° in the XRD curves were observed in the FD-SF preserved for 8 months, suggesting that there may have been some growth in SF crystallinity. The crystallinity of the FD-SF was calculated using MDI Jade6 software 6.5.26 by the FSD process (see Supplementary Materials Table S8). Figure 3D shows that the crystallinity slightly increased from 24.8% to 25.9% after 8 months of storage. This may be because the anhydrous environment induces the transition from amorphous to stable crystals [15]. The FTIR and XRD analyses reveal that there is no significant change in the conformation and aggregation structure of FD-SF after 8 months of storage.

### 3.5. Effect of Ultrasonication on FD-SF Solutions

**Figure 4:**
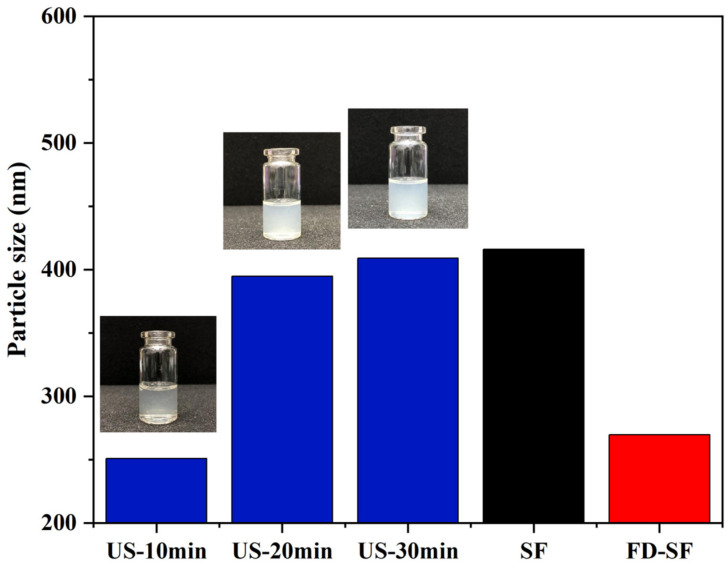
Particle size of SF solutions.

It is well known that MNs must have sufficient mechanical strength to penetrate the skin. Previous research suggested that the crystallinity of SF is positively correlated with its mechanical strength. Additionally, crystallinity is significantly improved during SF gelation accelerated by ultrasonication, which can be regulated for a duration of minutes to hours based on power output, time, and SF concentration [45,46]. The effect of ultrasonication on the FD-SF solutions was investigated by determining the particle size of the SF solutions at the same concentrations. In the preliminary experiment, we studied the effect of ultrasonic power on gelation time. When the ultrasonic power was 300, 200, or 150 W, the FD-SF solution gelled in 4, 8, and 10 min, respectively. More importantly, when the power exceeded 150 W, visible white particles appeared around the probe shortly after the ultrasound started, forming an agglomerated gel. Because of the localized white particles and agglomerated gel, the solution after sonication was inhomogeneous and unsuitable for MN preparation. When the sonication power was set to 100 W, the FD-SF gelled after 40 min but remained homogenous after 30 min, allowing for the investigation of the effect of different sonication durations on the mechanical strength of MNs. The power was then set to 100 W, potentially making the gelation process more controlled. The FD-SF solutions were ultrasonicated for 10, 20, and 30 min, with the times recorded as US-10 min, US-20 min, and US-30 min, respectively. As shown in Figure 4, the particle size of the regenerated SF solution was 416 nm, while the particle size of the FD-SF solution was reduced to 270 nm. The particle size slightly decreased after 10 min of ultrasonication, which could be due to ultrasonic fracture function. After 20 min, the particle size increased by 150 nm. It increased just a little between 20 and 30 min and approached the same size as the regenerated SF solution. After 30 min of sonication, the fluidity of the solution had greatly decreased. As the sonication time further extended, the solution completely transformed into a gel. Previous studies suggest that ultrasound can cause physical self-assembly cross-linking and β-sheet improved aggregation, resulting in larger particles [26,27]. However, the particle size will not grow endlessly. When the degree of cross-linking reaches saturation, particle size stops increasing. Since the sol–gel transition SF should be fluid to facilitate the preparation of MNs, the ultrasonic power and duration were set at 100 W and 20 min in the following experiments.

### 3.6. Effect of Ultrasonication on SF Structure in MNs

**Figure 5:**
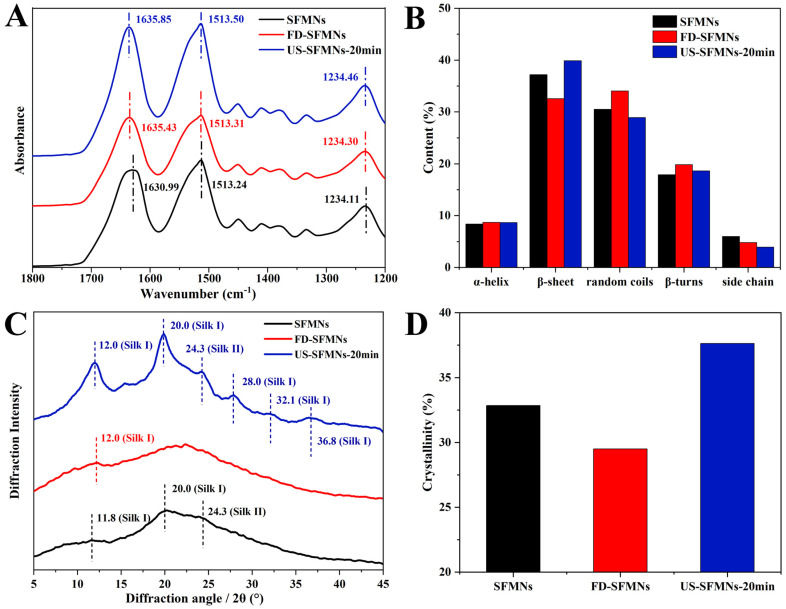
Structure analysis of SFMNs, FD-SFMNs, and US-SFMNs-20 min: (**A**) FTIR spectrum; (**B**) secondary structure content of SFMNs; (**C**) X-ray diffraction curve; (**D**) crystallinity.

The effect of ultrasound on SF structure in MNs was investigated by FTIR spectroscopy. The regenerated SF solution, as well as the FD-SF solution before and after ultrasonication (20 min), were fabricated into MNs and examined using FTIR spectroscopy. As shown in Figure 5, the three samples were identified as conventional SFMNs, FD-SFMNs, and US-SFMNs-20 min. Figure 5A shows FTIR peaks at 1630.99 cm^−1^, 1635.43 cm^−1^, and 1635.85 cm^−1^ for the conventional SFMNs, FD-SFMNs, and US-SFMNs-20 min, respectively. Figure 5B shows the FSD of the IR spectra using OMNIC software 9.2.86, which is specially designed to evaluate secondary structure content. The detail of the FSD process is also shown in the Supplementary Materials (Tables S5–S7). Figure 5B exhibits that both the conventional SFMNs and US-SFMNs-20 min had a β-sheet-dominated structures. Based on the comparison of Figure 5B and Figure 3B, the FD-SFMNs appeared to have higher β-sheet content than the FD-SF sponges. This demonstrates that the redissolution of the FD-SF sponges and the drying of the solution induced a random coil to β-sheet transition. As it is shown in Figure 5B, the β-sheet content of US-SFMNs is much greater at 40%, 7% higher than that of the FD-SFMNs and 2% higher than that of the conventional SFMNs. Previous studies have shown that increasing the β-sheet content of SF could enhance its mechanical strength [47,48,49], as the transition from random coil to β-sheet involves a crystallization process wherein the β-sheet is stacked to form microcrystals [50].

Figure 5C shows the XRD analysis of the conventional SFMNs, FD-SFMNs, and US-SFMNs. The FD-SFMNs have a broad peak in the XRD curves, with a minimal Silk I crystal peak at 12.0°. The US-SFMNs vary from the FD-SFMNs in that they have much sharper Silk I peaks at 12.0° and 20.0°, as well as a typical Silk I crystal peak at 28.0° and a Silk II peak at 24.3° [31,46,51]. The minimal peaks at 32.1° and 36.8° are also Silk I peaks [52]. Figure 5D shows that after ultrasonic modification, the crystallinity increases by 4.8% relative to the conventional SFMNs and 8.1% relative to the FD-SFMNs, which is consistent with the FTIR results. The details of the FSD process are shown in the Supplementary Materials (Table S9). The results demonstrated that ultrasonication of the FD-SF solution with 100 W of power for 20 min can increase the content of crystals in the US-SFMNs.

Previous studies have suggested that ultrasonication can reduce the gelation time of SF from weeks to minutes, which can be beneficial for SF hydrogel preparation. According to Wang et al.’s findings, sonicated SF solutions at 4%, 8%, and 12% (*w*/*v*) gelled within 0.5–2 h [25]. Bono et al. fabricated SF microgels by the ultrasonically induced gelation of SF in a water–oil emulsion phase [27]. In a rat femoral defect model, Diab et al. produced injectable SF hydrogels using ultrasonication [53]. For the first time, we prepared US-SFMNs with the intermediate state of sol–gel SF in our study by utilizing the sol–gel transition time window. The FTIR and XRD analyses demonstrate a β-sheet-rich structure (≈39.9% of the area of the amide I band) and high crystallinity (≈37.6%) of SF in the US-SFMNs, which can be beneficial for the preparation of MNs with superior mechanical properties.

### 3.7. Swelling and Dissolving Properties of SFMNs

**Figure 6:**
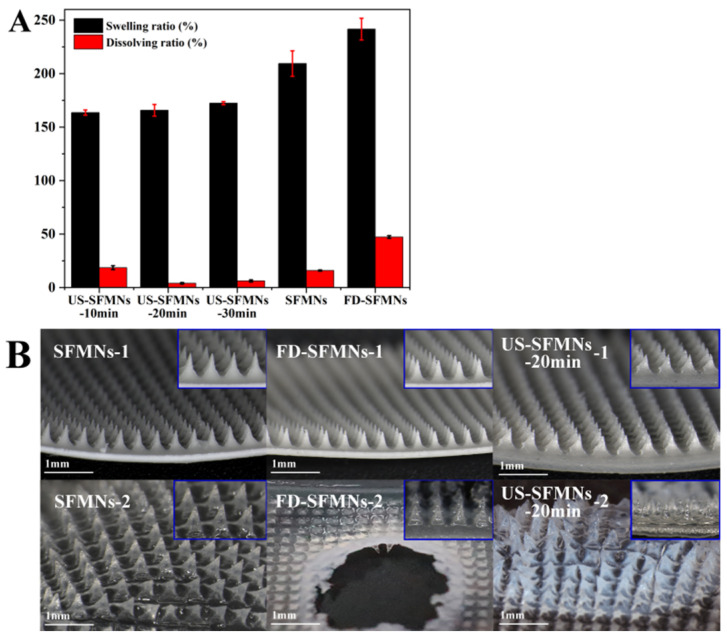
Swelling and dissolution properties of the SFMNs: (**A**) swelling and dissolving ratio; (**B**) the morphology of different SFMNs before (1) and after (2) PBS immersion.

The FD-SF solution was ultrasonicated for 10, 20, and 30 min and then prepared into MNs labelled as US-SFMNs-10 min, US-SFMNs-20 min, and US-SFMNs-30 min. The swelling and dissolving properties of the US-SFMNs were investigated. MNs prepared using the regenerated SF solution (conventional SFMNs) and freeze-dried SF solution (FD-SFMNs) were also examined as controls. The swelling and dissolving ratios of each MN are shown in Figure 6A. Compared to the conventional SFMNs, the swelling ratio of the FD-SFMNs increased by 32%, while the dissolving ratio increased to nearly 50%. The dissolving ratio of the US-SFMNs-10 min was 18.6%, somewhat higher than the conventional SFMNs but much lower than the FD-SFMNs. When the ultrasonic time was prolonged to 20 min, the dissolving ratio remarkably decreased to 4.0%. The results indicate that ultrasonic modification significantly reduced the dissolving ratio of the MNs. Figure 6B shows the morphology of the MNs before and after immersion in PBS. The central part of the FD-SFMNs dissolves in PBS after 5 min of immersion, which may result in a sudden drug release in the FD-SFMNs if loaded [12]. As shown in Figure 6B, the US- SFMNs-20 min had nearly no tip detachment after 24 h of immersion. The reason for the lower swelling and dissolving ratio of the US-SFMNs could be the increase in crystal content caused by ultrasonication, which is consistent with the results from the FTIR and XRD analyses. The ultra-low dissolving ratio of MNs can prevent needle material from remaining in the body, considerably increasing the safety of MN treatments.

### 3.8. Mechanical Strength and Insertion Ability of SFMNs

**Figure 7:**
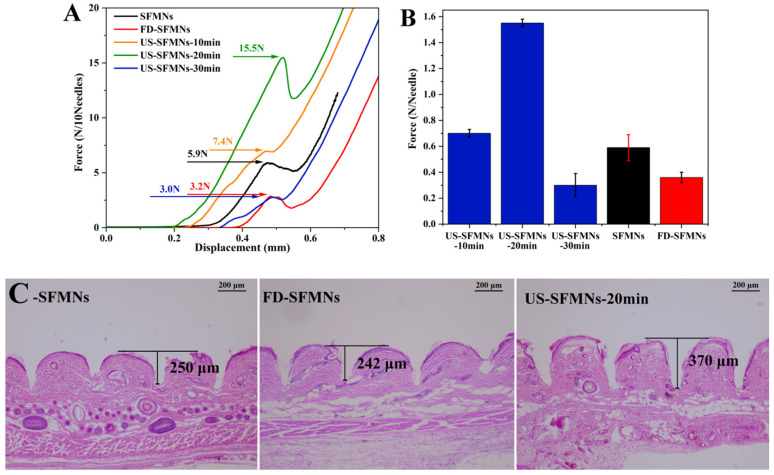
Mechanical properties and insertion ability of different SFMNs: (**A**) force–displacement curves; (**B**) compressive fracture force; (**C**) H&E staining image after insertion of SFMNs into rat skin.

The mechanical strength of the different SFMNs was determined by compression experiments using a texture analyzer. Figure 7A shows the force–displacement curves of different SFMN patches, including the conventional SFMNs, FD-SFMNs, and ultrasonically modified SFMNs (US-SFMNs-10 min, US-SFMNs-20 min, and US-SFMNs-30 min). Each SFMN has a peak in the curve, which is chosen as the fracture force point (labeled in Figure 7A). As shown in Figure 7B, the fracture force per needle of the conventional SFMNs was 0.59 N. For the FD-SFMNs, the fracture force decreased to 0.36 N per needle. The fracture forces of the US-SFMNs-10 min and US-SFMNs-20 min were found to be significantly greater than those of the conventional SFMNs. Notably, the US-SFMNs-20 min showed a fracture force of 1.55 N per needle, which was 2.6 times higher than the conventional SFMNs. However, prolonging the ultrasound duration to 30 min decreased the fracture force to 0.3 N per needle. The lower mechanical strength of the US-SFMNs-30 min compared to the US-SFMNs-20 min may be due to the poor fluidity of the SF solution after prolonged ultrasonication. Although ultrasonication can accelerate the sol–gel transition of SF from an aqueous solution to a β-sheet-rich hydrogel [54,55,56], insufficient fluidity may prevent the SF solution from entering the MN mold during the MN preparation. According to the findings from previous research, the minimum force required for MNs to penetrate through the stratum corneum is 0.045 N per needle [57], implying that the conventional SFMNs and FD-SFMNs can puncture the stratum corneum in theory. However, in practice, the mechanical strength of SFMNs needs to be higher to ensure skin epidermis penetration and lower the possibility of needle breaking [16]. Therefore, the greater mechanical strength of the US-SFMNs-20 min may assure the insertion, while allowing the MNs to penetrate deeper into the skin.

The insertion ability of the SFMNs was evaluated by H&E staining on rat skin treated with MNs ex vivo. The MNs were inserted into the rat skin using a force of 40 N for 30 s in vitro. The treated skin was sliced, H&E stained, and observed under a microscope. To achieve comparable results, the deepest part of the MN-created microhole must be sectioned. As a result, 20 slices were sectioned from each skin sample at the microhole, each with the same thickness. Meanwhile, each type of SFMN had three parallel skin samples. Figure 7C shows the representative H&E staining slices with the maximum hole depth for each SFMN. It shows that each needle could penetrate the upper layer of skin, the stratum corneum, which must be pierced for improving drug release [58]. The insertion depth of the conventional SFMNs was 250 μm, which is approximately 45% of the needle’s length. The US-SFMNs-20 min had the deepest depth of 370 μm, reaching 67% of the MN’s length. The results suggest that the US-modified SFMNs could penetrate deeper into the skin than the conventional SFMNs and FD-SFMNs, which is consistent with the mechanical strength findings.

Ultrasonication has been used to prepare silk films [56] and SF hydrogels [25,27,59]. SF molecular chains can undergo conformational changes from α-helix or random coil to β-sheet structure when stimulated ultrasonically. However, there have been few studies using ultrasonication to improve the mechanical strength of SFMNs. In this study, MNs were prepared using SF that was in an intermediate stage between solution and gel. On one hand, the modified SF solution controlled by appropriate ultrasonication parameters had sufficient fluidity to enter the MN mold for the MN preparation. On the other hand, ultrasonication could improve the β-sheet and crystal content of SF, leading to an increased mechanical strength and insertion ability of the modified SFMNs.

### 3.9. Enzymatic Degradation and Transdermal Permeation of SFMNs In Vitro

**Figure 8:**
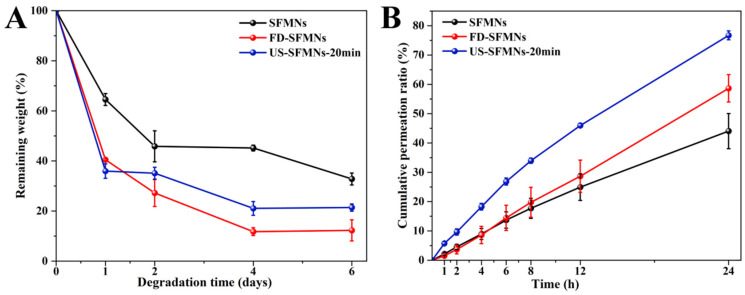
Enzymatic degradation and transdermal permeation of SFMNs in vitro: (**A**) enzymatic degradation in vitro; (**B**) transdermal permeation profiles of SFMNs in vitro.

The enzyme degradation of the different SFMNs was studied in vitro. Figure 8A shows the weight losses of the conventional SFMNs, FD-SFMNs, and US-SFMNs. The conventional SFMNs showed the lowest degradation. This may be due to the higher molecular weight of SF in the conventional SFMNs compared to the US-SFMNs and FD-SFMNs, which were prepared with the autoclaved SF solution with a molecular weight of SF 202 kD lower than the regenerated SF. According to earlier studies, the degradation rate of polymers was influenced by their molecular weight [60], with smaller molecular weight polymers degrading faster. Meanwhile, the US-SFMNs degraded more slowly than the FD-SFMNs upon exposure to the protease. This may be attributed to increased β-sheet crystals in the US-SFMNs. Previous research suggests that having more β-sheet crystals slows the degradation of SF [22]. Figure 8A shows that the conventional SFMNs degrade slower than the US-SFMNs, despite having lower β-sheet content. As the conventional SFMNs have higher-molecular-weight SF than the US-SFMNs, this implies that molecular weight might play a predominant role in this circumstance. Figure 8A indicates that the degradation rate was faster during the first two days, which may be caused by the noncrystalline silk in the SFMNs [61]. The degradation rate then decreased, possibly due to crystal degradation. In the presence of protease XIV, the remaining weight of the conventional SFMNs and US-SFMNs were 32.8% and 21.4% after 6 days, respectively. Despite the fact that further investigation is required to understand the in vivo degrading behavior of SFMNs, our findings suggest that the US-SFMNs may be a biodegradable transdermal delivery system capable of avoiding long-term skin irritation.

The in vitro transdermal profiles of a model drug, colchicine, from various SFMNs are displayed in Figure 8B. Table 1 shows the transdermal fluxes for each group. It shows that the conventional SFMNs and FD-SFMNs had comparable fluxes of 1.37 ± 0.29 and 1.32 ± 0.45 μg/(cm^2^∙h) for the first 4 h (*p* > 0.05). The flux of the conventional SFMNs then decreased to 1.22 ± 0.22 μg/(cm^2^∙h), while the flux of the FD-SFMNs slightly increased to 1.53 μg/(cm^2^·h) and remained constant during 4–24 h. This could be attributable to the higher dissolving ratio of the FD-SFMNs compared to the conventional SFMNs, resulting in faster drug release in the skin. The US-SFMNs had a higher transdermal flux than the FD-SFMNs and conventional SFMNs over the 24 h transdermal procedure. Furthermore, the US-SFMNs had a total permeation ratio of 76.7%, which was 1.7 and 1.3 times higher than the conventional SFMNs and FD-SFMNs, respectively. This could be due to the US-SFMNs’ higher mechanical strength and insertion capability, which might enable a wider contact area with the interstitial fluid of the skin and improve drug release. These results demonstrated that the US-SFMNs were more effective in transdermal permeation of colchicine.

Various approaches have been employed to improve the mechanical strength of SF-based MNs, including solvent vapor treatment and cross-linking [16,23]. Lu et al. used methanol vapor to increase the β-sheet content of SF [23]. Unfortunately, the treated SFMNs had a maximum fracture force of only 0.33 N per needle, despite a 24 h methanol vapor annealing time. A synergistic method of glutaraldehyde-based cross-linking and water vapor annealing post-treatment was employed Lin et al. [16]. When the two approaches were used simultaneously, the fracture force was 1.53 N per needle, which is comparable to the results of the US-SFMNs (1.55 N per needle) in our study. However, the poisonous glutaraldehyde residue is the issue that must be addressed. To the best of our knowledge, this is the first study to increase the mechanical strength of SF-based MNs through simple ultrasonication of the SF solution. The ultrasonically modified SF solution had sufficient fluidity for MN preparation, which could be achieved by optimization of the sonication parameters. The fracture force of the US-SFMNs was higher than the two-layered SFMNs with PVP as the baseplate in our earlier study [28]. H&E staining studies demonstrated that the US-SFMNs can penetrate the rat skin to a depth of 370 μm. Additionally, the US-SFMNs showed less dissolution in PBS than the conventional SFMNs. Moreover, the US-SFMNs had higher in vitro transdermal penetration and enzyme biodegradability compared to the conventional SFMNs. These findings demonstrate that the US-SFMNs have the potential for effective transdermal drug delivery.

## 4. Conclusions

In this study, solid SF material with long-term stability and complete and rapid redissolution was prepared by autoclaving and freeze drying the regenerated SF solution. The stability was confirmed by the FTIR and XRD analyses after 8 months of storage, which is beneficial for the development of SF-based MNs. For the first time, the SF solution was ultrasonically treated before MN preparation. Ultrasound at 100 W for 20 min resulted in an intermediate state of SF between the solution and hydrogel, with sufficient fluidity for MN preparation. The ultrasonically modified SFMNs (US-SFMNs) showed increased β-sheet content and crystallization, resulting in higher mechanical strength and insertion ability. Furthermore, the US-SFMNs demonstrated excellent transdermal penetration and enzymatic degradation in vitro. With appropriate swelling and low dissolution, the US-SFMNs have the potential to be a controlled-release MN system. Being a stable and water-soluble SF material, as well as having high mechanical strength and insertion capacity, make the US-SFMNs a promising transdermal drug delivery system.

## Figures and Tables

**Table 1:** Steady-state penetration rate of microneedles.

Group/[μg/(cm^2^·h)]	J_0–4 h_	J_4–12 h_	J_12–24 h_	Q_24h_ (μg/cm^2^)
SFMNs	1.37 ± 0.29	1.22 ± 0.22	0.97 ± 0.09	26.94 ± 3.66
FD-SFMNs	1.32 ± 0.45	1.53 ± 0.20	1.53 ± 0.11	35.86 ± 2.85
US-SFMNs-20min	2.79 ± 0.17	2.12 ± 0.04	1.57 ± 0.05	46.91 ± 0.92

## Data Availability

The original contributions presented in the study are included in the article/Supplementary Materials, further inquiries can be directed to the corresponding author.

## Supplementary Materials

The following supporting information can be downloaded at https://www.mdpi.com/article/10.3390/polym16223183/s1, Table S1: Vibrational band assignments in the amide I region; Table S2: Compression test parameters; Table S3: Deconvolution of the amide I bands of FD-SF; Table S4: Deconvolution of the amide I bands of FD-SF (8 months); Table S5: Deconvolution of the amide I bands of SFMNs; Table S6: Deconvolution of the amide I bands of FD-SFMNs; Table S7: Deconvolution of the amide I bands of US-SFMNs-20min; Table S8: Deconvolution of XRD spectra in silk I of FD-SF; Table S9: Deconvolution of XRD spectra in silk I of MNs.

## Nomenclature

SF	silk fibroin
MNs	microneedles
FD-SF	freeze-dried silk fibroin
FD-SFMNs	microneedles prepared with freeze-dried silk fibroin
US-SF	silk fibroin solution after ultrasonication
US-SF-10min	silk fibroin solution after 10 min of ultrasonication
US-SF-20min	silk fibroin solution after 20 min of ultrasonication
US-SF-30min	silk fibroin solution after 30 min of ultrasonication
US-SFMNs	microneedles prepared with ultrasonically treated silk fibroin
FSD	Fourier self-deconvolution
