# Supplementary material for: Ultrasonication-Induced Preparation of High-Mechanical-Strength Microneedles Using Stable Silk Fibroin
Source: Polymers (Basel). 2024 Nov 16;16(22):3183. doi: 10.3390/polym16223183 (PMC11598507; doi:10.3390/polym16223183)
Supplement: Supplementary file 1 [file polymers-16-03183-s001.zip › polymers-3278018-supplementary.pdf]

Supporting Information for

Ultrasonication-Induced Preparation of High-Mechanical-Strength Microneedles Using Stable Silk Fibroin

Huihui Liang [1], Jiaxin Chen [1], Guirong Qiu [1], Bohong Guo [1,2,*] and Yuqin Qiu [1,2,*]

1 Department of Pharmaceutics, School of Pharmacy, Guangdong Pharmaceutical University, Guangzhou 510006, China; 17703019860@163.com (H.L.); m13924254823@163.com (J.C.); qiugr0602@163.com (G.Q.)

2 Guangdong Provincial Key Laboratory for Research and Evaluation of Pharmaceutical Preparations, Guangdong Provincial Engineering Center of Topical Precise Drug Delivery System, Guangdong Pharmaceutical University, Guangzhou 510006, China

* Correspondence: guobohong@gdpu.edu.cn (B.G.); qiuyuqin@gdpu.edu.cn (Y.Q.)

Table of the contents:

1. Vibrational band assignments in the amide I region.
2. Compression test parameters.
3. Deconvolution of the amide I bands of FD-SF and SFMNs.
4. Deconvolution of XRD spectra in silk I of FD-SF and SFMNs.

1. Vibrational band assignments in the amide I region.

Table S1 Vibrational band assignments in the amide I region

Wavenumber range (cm^{-1})	Assignment
1605-1615	side chains
1616-1637	β-sheets
1638-1655	random coils
1656-1662	α-helices
1663-1696	β-turns
1697-1703	β-sheets

2. Compression test parameters.

Table S2 Compression test parameters

Test items	Compression test
Probe diameter	2 mm
Mode	Compression
Option	Return to start
Pre-test speed	0.1 mm/sec
Test speed	0.1 mm/sec
Post-test speed	10 mm/sec
Strain	90.0%
Trigger type	Auto（force）1 g
Data Acquisition Rate	500 pps

3. Deconvolution of the amide I bands of FD-SF and SFMNs.

Process the data using OMNIC software (Thermo Nicolet), first perform baseline correction and Fourier deconvolution, then Gaussian smoothing, and finally select a display range of 1605-1703cm^{-1}. Determine the scores of each secondary structure based on the peak area between different regions. Import data into Origin software (OriginLab) for plotting.

Table S3 Deconvolution of the amide I bands of **FD-SF**

Assignment	Wavenumber range (cm^{-1})	Area	Content
side chains	1605-1615	0.716	4.66%
β-sheets	1616-1637	3.798	24.72%
random coils	1638-1655	5.190	33.77%
α-helices	1656-1662	1.490	9.70%
β-turns	1663-1696	3.942	25.65%
β-sheets	1697-1703	0.231	1.50%

Table S4 Deconvolution of the amide I bands of **FD-SF (8 months)**

Assignment	Wavenumber range (cm^{-1})	Area	Content
side chains	1605-1615	0.552	4.95%
β-sheets	1616-1637	2.771	24.87%
random coils	1638-1655	3.751	33.66%
α-helices	1656-1662	1.114	10.00%
β-turns	1663-1696	2.804	25.16%
β-sheets	1697-1703	0.151	1.36%

Table S5 Deconvolution of the amide I bands of **SFMNs**

Assignment	Wavenumber range (cm^{-1})	Area	Content
side chains	1605-1615	0.902	6.00%
β-sheets	1616-1637	5.540	36.86%
random coils	1638-1655	4.587	30.52%
α-helices	1656-1662	1.261	8.39%
β-turns	1663-1696	2.690	17.90%
β-sheets	1697-1703	0.050	0.33%

Table S6 Deconvolution of the amide I bands of **FD-SFMNs**

Assignment	Wavenumber range (cm^{-1})	Area	Content
side chains	1605-1615	0.630	4.82%
β-sheets	1616-1637	4.610	32.21%
random coils	1638-1655	4.875	34.06%
α-helices	1656-1662	1.245	8.70%
β-turns	1663-1696	2.837	19.82%
β-sheets	1697-1703	0.053	0.37%

Table S7 Deconvolution of the amide I bands of **US-SFMNs-20min**

Assignment	Wavenumber range (cm^{-1})	Area	Content
side chains	1605-1615	0.824	3.91%
β-sheets	1616-1637	8.339	39.52%
random coils	1638-1655	6.103	28.92%
α-helices	1656-1662	1.826	8.65%
β-turns	1663-1696	3.932	18.64%
β-sheets	1697-1703	0.076	0.36%

4. Deconvolution of XRD spectra in silk I of FD-SF and SFMNs.

The crystallinity was quantitatively quantified using MDI Jade software (International Centre for Diffraction Data). Firstly, use smoothing tools to process the curve, and then perform baseline correction to fit the curve peaks. Finally, obtain the crystallization report, select Silk I, and obtain the relative areas and contents of each crystal. Import data into Origin software (OriginLab) for plotting.

Table S8 Deconvolution of XRD spectra in silk I of **FD-SF**

Substance	Center/°	Area%	Content
FD-SF	19.7	20.0	24.77%
FD-SF (8 months)	19.3	8.58	13.19%
	23.2	8.28	12.72%

Table S9 Deconvolution of XRD spectra in silk I of **MNs**

Substance	Center	Area%	Content
SFMNs	11.8	6.5	10.31%
	20.0	14.2	22.54%
FD-SFMNs	12.0	3.8	29.51%
	12.0	1.9	1.90%
US-SFMNs-20min	20.0	34.8	34.83%
	28.0	0.9	0.90%
